# The Role of Vitamin D in Small Animal Bone Metabolism

**DOI:** 10.3390/metabo10120496

**Published:** 2020-12-03

**Authors:** Rafael Vessecchi Amorim Zafalon, Bruna Ruberti, Mariana Fragoso Rentas, Andressa Rodrigues Amaral, Thiago Henrique Annibale Vendramini, Fernanda Chicharo Chacar, Marcia Mery Kogika, Marcio Antonio Brunetto

**Affiliations:** 1 Pet Nutrology Research Center, Nutrition and Production Department, School of Veterinary Medicine and Animal Science, University of São Paulo, Jardim Elite, Pirassununga 13635-900, Brazil; rafael.zafalon@usp.br (R.V.A.Z.); mariana.rentas@usp.br (M.F.R.); thiago.vendramini@usp.br (T.H.A.V.); 2 Small Animal Internal Medicine Service, Veterinary Teaching Hospital, School of Veterinary Medicine and Animal Science, University of São Paulo, Cidade Universitária, São Paulo 05508-270, Brazil; brunaruberti@usp.br (B.R.); mmkogika@usp.br (M.M.K.); 3 Veterinary Nutrology Service, Veterinary Teaching Hospital, School of Veterinary Medicine and Animal Science, University of São Paulo, Cidade Universitária, São Paulo 05508-270, Brazil; andressa.rodrigues.amaral@usp.br; 4 Department of Internal Medicine, Federal Institute of Education, Science and Technology of South of Minas Gerais, IFSULDEMINAS, Muzambinho 37890-000, Brazil; fernanda.chacar@muz.ifsuldeminas.edu.br

**Keywords:** 1,25(OH)_2_D_3_, 25(OH)D, calcium, cat, dog, nutrition, osteomineral homeostasis, phosphate

## Abstract

Dogs and cats have differences in vitamin D metabolism compared to other mammalian species, as they are unable to perform vitamin D cutaneous synthesis through sun exposure. Therefore, they are dependent on the dietary intake of this nutrient. The classic functions of vitamin D are to stimulate intestinal calcium and phosphate absorption, renal calcium and phosphate reabsorption and regulate bone mineral metabolism. Thus, it is an important nutrient for calcium and phosphorus homeostasis. This review highlights the evidence of the direct and indirect actions of vitamin D on bone mineral metabolism, the consequences of nutritional imbalances of this nutrient in small animals, as well as differences in vitamin D metabolism between different size dogs.

## 1. Introduction

Vitamin D is an essential nutrient for dogs and cats since they are unable to synthesize vitamin D_3_ through skin sun exposure; thus, it is essential that they receive this nutrient in their diet [1,2]. vitamin D has two nonactive forms in nature: cholecalciferol or vitamin D_3_, and ergocalciferol or vitamin D2. After ingestion, vitamin D undergoes a multistep enzymatic conversion until conversion to an active vitamin known as 1,25-dyhydroxyvitamin D (1,25(OH)_2_D_3_) [3]. After activation, vitamin D binds to the vitamin D receptor (VDR), a representative receptor for the steroid receptors family, which mediates its biological functions [4] that go beyond the traditional role of calcium and phosphate homeostasis [5].

Regarding bone metabolism vitamin D functions, calcitriol target organs are intestine, kidneys, bone tissue and the parathyroid gland [6,7]. In the intestine, calcitriol acts on transcellular and passive paracellular calcium transport besides phosphate absorption [8,9]. In the kidneys, calcitriol stimulates renal calcium and phosphate reabsorption. Finally, calcitriol acts in the parathyroid gland regulating parathyroid hormone (PTH) secretion. All these functions can indirectly regulate bone growth and mineralization. In addition, it is known that both osteoblasts and chondrocytes express the enzyme 1α-hydroxylase and VDR [10] and it has been demonstrated that vitamin D can act directly on bone growth and mineralization [11,12,13], as well as in bone remodeling [14].

Although the importance of vitamin D in bone metabolism is well established in humans, its deficiency is prevalent worldwide [15]. In dogs and cats, with the wide use of complete and balanced commercial foods, there was a decrease in vitamin D deficiency cases diagnosis [16], which was very common in the past. However with the growing interest in homemade pet foods, there is a growing risk of hypovitaminosis D [17,18].

It is known that vitamin D deficiency can result in hypocalcemia and hypophosphatemia and, consequently, can result in bone disease characterized by impaired bone mineralization associated either with rickets or osteomalacia [7,16]. In addition, dietary vitamin D deficiency can result in both direct and indirect nutritional secondary hyperparathyroidism (NSHP) [7,19,20]. Excessive vitamin D is also harmful and causes of hypervitaminosis D in small animals. This can be due to commercial food formulation miscalculation [21,22,23,24], accidental consumption of rodenticides [25,26,27,28,29], consumption of certain plants that may contain calcitriol glycosides (such as jessamine [30]) and the use of topical ointments for psoriasis treatment, which are based on vitamin D analogs such as calcipotriol and maxacalcitol [31,32,33,34]. Acute vitamin D intoxication results in hypercalcemia, which causes clinical signs such as polydipsia, polyuria, anorexia, vomiting, constipation, seizures, etc. In the case of chronic intoxication, osteochondrosis and delayed bone remodeling can occur mostly in growing giant breed dogs [35,36,37].

Therefore, this review aims to summarize the current knowledge about the direct and indirect role of vitamin D in bone metabolism, as well as discussing the negative effects of vitamin D deficiency and excess in dogs’ and cats’ bone health.

## 2. Vitamin D Metabolism in Small Animals

Two forms of vitamin D are found in nature, cholecalciferol or vitamin D_3_, and ergocalciferol or vitamin D2. While ergocalciferol is synthesized from ergosterol found in fungi and plants through exposure to UVB ultraviolet rays (290–315 nm) [38], cholecalciferol is synthesized in the skin of several mammalian animals after sun exposure [39,40]. In the deep layers of the epidermis (spinous and basal strata) the precursor of cholecalciferol, 7-dihydrocholesterol (a derivative of cholesterol), is located in the cell membranes’ phospholipid bilayer [41]. The 7-dihydrocholesterol absorbs a UVB photon and this allows the photolytic break of the bond between carbons nine and 10 of ring B of the pentanoperhydrophenanthrene cycle, forming previtamin D_3_, which has two broken rings. This substance is thermostable and undergoes heat-induced isomerization giving rise to vitamin D_3_, which has a special, more stable, configuration. Vitamin D_3_ gains circulation due to the steric energy of this new three-dimensional conformation of the molecule [41].

Dogs and cats are unable to perform cutaneous synthesis of Vitamin D_3_ through sun exposure, due to the high activity of 7-dihydrocholesterol-Δ7-reductase enzyme that converts 7-dehydrocholesterol into cholesterol. Therefore, a significant decrease in skin 7-dehydrocholesterol concentrations occurs [3,39,40]. In an in-vitro study conducted by How et al. [39], total 7-dehydrocholesterol concentrations in the skin of dogs and cats before sun exposure, were, on average, almost 10 times lower when compared to rats. After sun exposure, there was no change in vitamin D_3_ concentrations in dog and cat skin extracts, while there was a 40-fold increase in vitamin D_3_ concentrations in rat skin. In cats, an in-vivo study demonstrated that kittens with low vitamin D status upon receiving a 7-dihydrocholesterol-Δ7-reductase enzyme inhibitor had a progressive increase in vitamin D status when exposed to ultraviolet light for 2 h/day, even if receiving a purified vitamin D-free diet, while a group that did not receive the enzyme inhibitor had no change in vitamin D status [40]. These authors demonstrated that the high activity of 7-dihydrocholesterol-Δ7-reductase prevents adequate vitamin D_3_ skin synthesis.

The inability to synthesize cutaneous vitamin D_3_ justifies the fact that no seasonal variation in vitamin D status was observed in dogs in a study conducted in Australia [42]. It is believed that the inability to synthesize vitamin D_3_ in the skin in dogs and cats is related to the evolutionary aspect that once, in nature, these animals consumed preys which stores vitamin D_3_ in the liver and adipose tissue. Therefore, when prey was consumed regularly, their vitamin D requirements were met. Consequently these animals did not need to develop the ability of cutaneous vitamin D_3_ synthesis [3,43].

As a consequence, dogs and cats are totally dependent on their dietary intake. After ingestion, both cholecalciferol and ergocalciferol are absorbed in a process that depends on digestive enzymes, bile acids and chylomicrons [44]. After absorption, cholecalciferol and/or ergocalciferol are transported through intestinal lymphatic vessels and the portal system, linked to a glycoprotein vitamin D binding protein (VDBP), to the liver where they undergo a hydroxylation process on Carbon 25, which can occur through enzymes of the cytochrome P450 family such as CYP27A1, CYP3A4, CYP2R1 and CYP2J3 [45], also known as 25-hydroxylases, forming 25-hydroxyvitamin D (25(OH)D) or calcidiol. If the animal has high circulating calcidiol concentrations, the absorbed cholecalciferol can be stored in adipose tissue and, to a lesser extent, in muscle tissue, instead of being transported for hydroxylation in the liver [46].

Dogs convert ergocalciferol to calcidiol as well as cholecalciferol [44]. However, cats use cholecalciferol more efficiently [47]. This may be due to the type of food that these animals had in nature during the evolutionary process. Dogs have a more omnivorous nature, so that in nature they consumed ergocalciferol and then developed the ability to use this form of vitamin D to synthesize calcidiol in the liver. In contrast, cats are considered strict carnivores, and in nature they did not consume ergocalciferol, a fact that may justify their lesser ability to use this substance for hepatic calcidiol synthesis [3]. After hepatic synthesis, calcidiol is transported by VDBP to the proximal renal tubules where it undergoes a hydroxylation process in carbon 1 through a mitochondrial enzyme of the CYP450 family, 1α-hydroxylase (CYP27B1), giving rise to 1,25 dihydroxyvitamin D [1,25(OH)_2_D] or calcitriol, the active metabolite of vitamin D which performs all its known hormonal actions. It is worth mentioning that renal synthesis is largely responsible for circulating calcitriol. However, it has been demonstrated in humans that several other tissues express 1α-hydroxylase, including the breast, parathyroid gland, colon, prostate, beta-pancreatic cells, placenta, brain, immune cells, endothelial cells and keratinocytes [48].

Although calcitriol is the active metabolite, vitamin D status is assessed by measuring circulating calcidiol concentrations as it has a long biological half-life, ranging from 10 days to three weeks, whereas the biological half-life of calcitriol varies from three to six hours [49]. Besides, calcidiol circulates in concentrations around 1000 times higher compared to calcitriol. It is also worth mentioning that calcitriol has a more hydrophobic nature, which makes it difficult to measure [3,50], in addition to it being less stable [7,51].

## 3. Regulation of Calcitriol Synthesis and Catabolism

Calcitriol synthesis regulation is influenced by several factors, such as plasma calcium and phosphate concentrations, parathyroid hormone (PTH), fibroblast growth factor (FGF-23), klotho gene and calcitonin [3,52,53,54,55]. In hypocalcemic conditions, there is an increase in PTH release, which can directly stimulate the activity of 1α-hydroxylase by stimulation of the 1α-hydroxylase promoter gene, or indirectly by stimulating 1α-hydroxylase mRNA expression and then the activity of 1α-hydroxylase via cAMP-dependent pathway in order to increase calcitriol synthesis [7,56,57,58]. Under normocalcemic conditions, calcitonin acts to maintain serum calcitriol concentrations, by inducing 1α-hydroxylase transcription in renal cells, which seems to occur due to positive regulation of the transcription factor C/EBPβ [55].

The 24-hydroxylase enzyme is responsible for vitamin D catabolism. This mitochondrial enzyme is found mainly in proximal renal tubules but it is also expressed in intestine, keratinocytes, fibroblasts, lymphocytes and macrophages [59]. The 24-hydroxylase enzyme acts through two metabolic pathways and promotes hydroxylation in the side carbon chain, both in calcidiol and calcitriol. It promotes hydroxylation on carbon 23 of calcitriol, producing 1α,23,25-trihydroxyvitamin D_3_ (1α,23,25(OH)_3_D_3_) which, in turn, undergoes an oxidation process giving rise to 1α,25-dihydroxyvitamin D_3_ -26,23-lactone.

Through a second metabolic pathway, 24-hydroxylase promotes hydroxylation on carbon 24 of calcidiol and calcitriol, resulting in the formation of 24,25–dihydroxyvitamin D_3_ (24,25(OH)_2_D_3_) and 1α,24,25-trihydroxyvitamin D_3_ (1α,24,25(OH)_3_D_3_), respectively. The 1α,24,25(OH)_3_D_3_ still undergoes an oxidation process, forming calcitroic acid, a metabolite excreted through bile [60,61,62]. These metabolites functions are not yet well known, but 24,25(OH)_2_D_3_ is believed to be related to the bone mineralization process [63].

Calcitriol stimulates 24-hydroxylase activity, playing a protective role against hypercalcemia [61]. In situations of high serum phosphate concentrations, there is an increase in synthesis and release of FGF-23, an important peptide produced by osteoblasts and mainly by osteocytes, with hypophosphatemic action, so that it stimulates renal phosphorus excretion, increases 24-hydroxylase activity, decreases 1α-hydroxylase activity and, therefore, decreases calcitriol synthesis, since this hormone has the function of intestinal phosphorus absorption and tubular reabsorption of inorganic phosphate [61,64,65]. Calcitriol synthesis regulation is also affected by a transmembrane protein called Klotho, which is a mandatory and necessary coreceptor for FGF-23 to bind to its receptor [66].

## 4. Differences in Vitamin D Metabolism between Miniature and Giant Dog Breeds

It is known that dogs of giant breeds are prone to develop skeletal disorders when compared to dogs of small breeds fed with low or excessive calcium diets [67]. Among other things, this seems to be related to differences in vitamin D metabolism. In the study by Hazewinkel and Tryfonidou [68], the plasma concentrations of the vitamin D main metabolites were measured in giant breed dogs (adult weight: 60 kg) and small breed dogs (adult weight: 6 kg) at 14 weeks of age managed under the same conditions. There was no difference in concentrations of 25(OH)D and 1,25(OH)_2_D_3_. However giant breed dogs had lower 24,25(OH)_2_D concentrations, with values which were about 10 times lower than small breed dogs.

In another study, carried out by Tryfonidou et al. [67], Great Dane puppies showed higher plasma 1,25(OH)_2_D concentrations and lower plasma 24,25(OH)_2_D concentrations compared to miniature poodle puppies. According to Hazewinkel and Tryfonidou [68], the lower plasma 24,25(OH)_2_D concentrations may be related to disturbances in endochondral ossification during the rapid growth phase observed in large and giant breed dogs. Osteochondrosis is a disorder of maturation that results in a delay of cartilage mineralization. It is known that both 24,25(OH)_2_D and 1,25(OH)_2_D play an important role in differentiating cartilage cells and matrix mineralization [68,69]. In order to avoid disturbances in endochondral ossification, the physiological balance between 24,25(OH)_2_D and 1,25(OH)_2_D is of major importance [70]. Therefore, the imbalance between these vitamin D dihydroxylated metabolites in large and giant breeds dogs seems to be related to a greater disposition for osteochondrosis during growth.

The reason why giant breed dogs have lower plasma 24,25(OH)_2_D concentrations is not yet fully elucidated, but it is believed that this can be explained by higher circulating growth factor concentrations that these animals present, such as growth hormone (GH) and insulin-like growth factor I (IGF-I) [68]. Even in the study by Tryfonidou et al. [67], higher plasma GH and IGF-1 concentrations were observed in Great Dane puppies than in miniature poodle puppies. In humans, an increase in circulating 1,25(OH)_2_D concentrations has been reported after GH therapy in children with GH deficiency [71], normal adults [72] and elderly people [73], and Condamine et al. [74] observed that IGF-1 stimulates the 1,25(OH)_2_D synthesis in mammalian renal cells under conditions of hypophosphatemia. In addition, an association has been demonstrated between high plasma GH and IGF-I concentrations and low plasma 24,25(OH)_2_D concentrations [75], as well as showing that GH and IGF-I decrease 24-hydroxylase activity [76], which may justify this association.

## 5. The VDR Receptor

Brumbaugh and Haussler [77] first described the vitamin D receptor, a nuclear transcription factor belonging to a nuclear receptor superfamily for steroid hormones [3,78,79]. For the biological activity of vitamin D to occur, circulating calcitriol bound to VDBP crosses the cell membrane and binds to VDR. After this binding, a calcitriol-VDR complex is formed which interacts with retinoic acid receptor (RXR) forming a calcitriol-VDR-RXR heterodimer that acts on promoter regions of target genes, known as vitamin D response elements (VDRE) [80,81]. Consequently, there is a cascade of molecular interactions evolving nuclear coactivators and transcription factors, positive and negative, which interact with calcitriol-VDR-RXR regulating the transcription and/or suppression of specific genes [6,79,82]. Under gene induction or repression, changes in the expression of proteins required for a biological response occurs [83,84]. This vitamin D genomic response process may involve regulation of about 2000 genes, which represent almost 10% of the human genome [85].

The VDR can also show nongenomic responses, which occurs when calcitriol interacts with VDR located in sphingolipid and cholesterol rich cell membranes [86]. In response to binding of calcitriol to these receptors, there is an activation of guanylate cyclase, phosphatidylinositol-3-kinase (PIK 3) [87], cyclic adenosine monophosphate (cAMP), mitogen-activated protein kinase (MAPK) [88]; protein kinase (PK) A and phospholipase C (PLC) pathways [89], the opening of voltage-dependent calcium and chlorine channels [89,90] and mobilization of calcium from the sarcoplasmic reticulum [89,91]. According to Norman et al. [89], these mechanisms cause the migration of smooth muscle cells from the endothelium, rapid absorption of calcium by the duodenal epithelium and exocytosis of insulin by β-pancreatic cells.

## 6. The Functions of Vitamin D Related to Bone Metabolism

Regarding vitamin D functions related to bone metabolism, the target organs of calcitriol are intestine, kidneys, bone tissue and parathyroid gland [6,7].

### 6.1. Effects of Vitamin D on the Intestine

In the intestine, calcitriol acts on both transcellular and passive paracellular calcium transport [8,9]. Transcellular calcium transport consists of three stages: calcium entry into the enterocyte through the calcium epithelial channel transient receptor potential vanilloid type 6 (TRPV6); the cytoplasmic transfer of calcium linked to the calbindin-D9k protein, which facilitates the diffusion of calcium through the cell and the extrusion of calcium through the basolateral membrane via Ca^2+^–ATPase (PMCA 1b) and/or a Na-Ca exchanger [8], which results in calcium entering the circulation. Vitamin D positively regulates TRPV6 and calbindine-D9k and, consequently, there is an increase in calcium absorption [92,93]. The importance of vitamin D in regulation of TRPV6 and calbindin-D9k was proven in studies with VDR knockout mice in which a reduction of 50% in intestinal calbindin-D9k mRNA was observed, as well as a reduction of up to 95% in TRPV6 mRNA, when compared to wild type mice [94,95].

Paracellular transport occurs mainly in the most distal regions of the intestine, but it can happen over the entire length of the intestine [96]. This pathway consists of a luminal electrochemical gradient and the integrity of the intercellular junctions [96,97]. The tight junctions are located between apical and basolateral membranes of the enterocyte, providing cell-cell adhesion in epithelial and endothelial cell layers. These junctions provide a primary barrier to the passage of ions, proteins, cytoskeleton molecules and cytoplasmic signaling molecules [97]. There are several transmembrane components at the tight junctions, such as sodium/potassium ATPase, claudin 3, aquaporin 8, cadherin 17 and RhoA, which can be suppressed by calcitriol and suggests that vitamin D regulates the permeability of tight junctions and, consequently, paracellular calcium transport [98]. In addition, calcitriol stimulates the expression of paracellins, intercellular proteins that form channels where calcium is passively transferred due to differences in concentration gradient [92].

In addition to calcium absorption, in the intestine, vitamin D also acts on phosphate absorption. Calcitriol stimulates phosphate absorption via the Naþ phosphate transporter (NaPi-IIb) pathway so that calcitriol increases Na-Pi transporter expression, which corresponds to a family of SLC34 solute carriers (SLC34A2) [99].

### 6.2. Effects of Vitamin D on the Kidneys

Calcitriol stimulates renal calcium reabsorption so that calcium transport through renal epithelial cells is similar to calcium transport through intestinal epithelial cells. In the apical cell membrane of the distal convoluted tubules and collecting ducts there is an epithelial calcium channel (TRPV5) that is responsible for transporting calcium into the cells. Calcium transport occurs through renal tubular cells through the calbindin-D28K protein, and in the basolateral cell membrane there are calcium transporters PMCA1b and NCX responsible for transporting calcium into the bloodstream [100]. In addition, a calcium influx protein called epithelial Ca21 channel (ECaC) was discovered. ECaC acts in the active reabsorption of calcium in the distal portion of the nephron [101,102,103]. Calcitriol upregulates TRPV5, calbindin-D28K, PMCA1b, NCX1 and ECaC gene expression [7,104,105,106].

### 6.3. Effects of Vitamin D on the Parathyroid Gland

It is known that calcitriol receptor mRNA has high expression in the parathyroid gland [107,108]. After the discovery of the active form of vitamin D, the effects of this hormone on the parathyroid gland began to be investigated and it was demonstrated in-vitro that calcitriol decreased PTH production in primary bovine parathyroid cell culture by decreasing the PTH gene transcription [109]. Subsequently, calcitriol was shown to decrease levels of PTH mRNA in rats [90]. Because of the effect of reducing PTH synthesis in humans with chronic kidney disease (CKD), calcitriol or analogues have been used to treat secondary renal hyperparathyroidism [110]. In contrast, in a study conducted by Hostutler et al. [111] with healthy and CKD cats, no difference in serum PTH concentrations was observed after a 14-day oral treatment with calcitriol (2.5 ng/kg every 24 h or 8.75 ng/kg every 84 h), and no difference in serum ionized calcium concentrations was noticed. It is worth mentioning that further studies are needed to investigate whether the dosages used in that study were insufficient to reduce PTH synthesis. According to International Renal Interest Society (IRIS) [112], there is evidence to suggest that the rational use of calcitriol (1.5 to 3.5 ng/kg) prolongs the survival of dogs with CKD in stages three and four, where phosphate is controlled and ionized calcium and PTH are monitored. As for cats, according to IRIS [113], the beneficial effects of an ultralow dose of calcitriol have not yet been established, so its use is not currently recommended.

In humans, the treatment with calcitriol in CKD patients promotes beneficial effects on the kidneys unrelated to PTH and mineral metabolism, including activity of renin-angiotensin-aldosterone system suppression, reduction of proteinuria, reduction of podocyte loss associated with glomerular hypertrophy and antiproliferative and antifibrotic effects [114,115,116,117]. These effects seem to be important to explain the benefits demonstrated by calcitriol therapy in CKD human patients, where it has been shown to limit the progression of CKD, improve survival and be independently associated with preservation of renal function [116,118].

The mechanisms by which vitamin D decreases PTH synthesis are well described, so that it suppresses the parathyroid gland cells growth gland by decreasing growth factors (transforming growth factor—epidermal growth factor receptor growth loop) and through the increase in inhibitors of cell growth (cyclin-dependent kinase inhibitors p21 and p27) [7,19]. In addition, calcitriol has been shown to positively regulate the transcription of gene encoding the calcium-sensing receptor (CaSR) in the parathyroid gland, which provides greater sensitivity of this gland to plasma ionized calcium concentration resulting in decreased PTH secretion [119].

### 6.4. Effects of Vitamin D on Bone Remodeling

In bone tissue, under conditions of hypocalcemia, calcitriol works in conjunction with PTH to mobilize calcium from bones for circulation to increase circulating ionized calcium concentrations [12,120]. This occurs through the RANKL/osteoprotegerin (OPG) regulatory system, so that in immature osteoblastic cells calcitriol stimulates the activator receptor for NF-kB ligand (RANKL), increasing osteoclastogenesis [14]. RANKL binds to osteoclasts via the activator receptor for NF-kB (RANK) or via the osteoprotegerin receptor [121]. The binding of RANKL to RANK induces differentiation and maturation of osteoclast progenitor cells to osteoclasts. On the other hand, the binding of RANKL to osteoprotegerin inhibits osteoclastogenesis and bone resorption. Calcitriol inhibits osteoprotegerin production, thus allowing bone resorption [122,123,124]. Therefore, VDR signaling negatively regulates bone mass, which was demonstrated in a study with mice with inactivated VDR in which an increase in bone mass was observed [125].

### 6.5. Effects of Vtamin D on Bone Growth and Mineralization

In the past, it was believed that calcitriol influenced bone growth and mineralization only indirectly, that is, by increasing the calcium and phosphorus concentrations in the circulation through stimulating intestinal absorption and renal reabsorption of these minerals [126]. Later, it was noticed that both osteoblasts and chondrocytes express 1α-hydroxylase and VDR [10]. In addition, an aberrant growth plaque development was observed in VDR-deficient mice before the appearance of hypocalcemia, which reinforces the possible defined role by 1,25(OH)_2_D-mediated signaling in the growth of endochondral bone [11].

As previously discussed, in negative calcium balance, VDR signaling is directed towards maintaining circulating calcium concentrations, that is, there is an increase in resorption and a decrease in bone mineralization [127]. In contrast, in situations of positive calcium balance, the role of VDR signaling in bone tissue is still unclear. However, it is believed that specific functions depend on osteoblast differentiation stages [127]. In mature osteoblasts, calcitriol, via VDR signaling, has been shown to perform anabolic and anticatabolic activity, consequently promoting an increase in bone mass as demonstrated in a study with mice with transgenetically increased VDR expression [12]. In this study, it was observed that the decrease in bone resorption occurred due to increased expression of osteoprotegerin, which resulted in a decrease in the RANKL/osteoprotegerin ratio. In addition, it was shown that calcitriol can induce Lrp5 mRNA transcripts (Lrp5 acts as a coreceptor in Wnt signaling [13]) so it is important in bone formation and mineralization.

## 7. Vitamin D Recommendations for Small Animals

Minimum and maximum recommendations of cholecalciferol for dogs and cats according to National Research Council (NRC) [1], European Pet Food Industry Federation (FEDIAF) [128] and Association of American Feed Control Officials (AAFCO) [129] are presented in Table 1 and Table 2.

Few studies have investigated vitamin D requirements for dogs and cats, and all of these studies were performed in puppies and kittens since the demand for skeletal calcification is higher at this life stage and growing animals are more sensitive to vitamin D deficient diets. As there are no studies that investigated vitamin D requirements for adult dogs and cats, as well as breeding females, the recommendations are based on studies of growing animals.

A pioneering study carried out in 1918 by Mellanby [130] observed that the use of cod liver oil and butter included in food for dogs with rickets improved clinical condition of these animals. The first study to work with doses was published in 1939 by Arnold and Elvehjem [131]. These authors performed two experiments with Great Dane puppies, suggesting a dosage of 20 IU vitamin D per 100 g food of about 3.8 kcal g^−1^ (212 IU kg^−1^ diet of 4 kcal g^−1^) for growing puppies consuming a diet with 9.0 g of calcium and 7.5 g of phosphorus per kg. However, it was only in 1944 that the first vitamin D recommendation for dogs was proposed by Michaud and Elvehjem [132], in which the authors recommended an intake of 10 IU to 20 IU kg BW^−1^·d^−1^. Finally, Tryfonidou [133] provided a diet with 500 IU of cholecalciferol.kg^−1^ to Great Dane puppies and did not observe any adverse bone disease.

Regarding cats, few studies have evaluated different concentrations of vitamin D in feline nutrition. The first study was published by Gershoff et al. [134] in which the authors performed oral supplementation on kittens with 250 IU of cholecalciferol per animal twice a week, and prevention of rickets was observed in these animals. In addition, the authors suggested that adult cats have a lower vitamin D requirement since they observed remission of clinical signs in cats older than 12 months who were fed diets with vitamin D inadequacies.

Morris et al. [135] evaluated six groups of kittens and each group received a different cholecalciferol concentrations diet (0.0, 120, 125, 250, 500, 750 or 1000 IU kg^−1^ of diet). In this study, the authors provided low cholecalciferol concentration diets to the kittens’ mothers during pregnancy and lactation, thus inducing a reduction in the vitamin D kitten reserves. The kittens were fed from nine to 22 weeks of age with the diets and it was noticed that the plasma 25(OH)D concentrations grew linearly with dietary intake of cholecalciferol. In addition, animals fed 0.0 and 125 IU cholecalciferol kg^−1^ of diet, consumed the food until 34 weeks of age. In this part of the study, the authors observed a sharp drop in the plasma 25(OH)D concentrations of the animals that consumed the food without the addition of vitamin D. However, the kittens that consumed the food with 125 IU cholecalciferol kg^−1^ of diet had higher plasma 25(OH)D concentrations at 22 and 34 weeks than at nine weeks of age, which indicates that the inclusion of 125 IU cholecalciferol kg^−1^ of diet would be suitable for kittens. However, the authors recommended a dose of 250 IU cholecalciferol kg^−1^ of diet to maintain a safety margin for both kittens, pregnant and lactating cats.

Morris and Earle [136] published another study with growing kittens, using a concentration of 125 IU cholecalciferol kg^−1^ of purified diet, with calcium concentrations ranging from 3.8 to 8.1 g/kg of diet and constant Ca:P ratio. In this study, the authors also observed plasma 25(OH)D concentrations within the normal range.

## 8. Vitamin D Deficiency in Dogs and Cats

Vitamin D deficiency can be caused by abnormal vitamin D metabolism related to congenital abnormalities due to genetic mutations [137,138], as well as by the intake of unbalanced diets [7,18]. Currently, in the face of the growing production of commercial complete foods, dietary vitamin D deficiency has become less common in dogs and cats [16]. However, the recent increased use of unbalanced diets, that may include homemade and raw foods or inappropriate feeding strategies used by veterinarians or pet owners, may increase nutritional disorders in animals, including vitamin D deficiency [17,18].

As previously discussed, vitamin D requirements are not well established for dogs and cats, which are dependent on vitamin D dietary intake. Vitamin D deficiency results in hypocalcemia and hypophosphatemia, which can result in clinical manifestation of bone disease characterized by impaired bone mineralization associated either with rickets or osteomalacia [7,16]. Rickets is a disease characterized by delayed mineralization of growth cartilage and a failure to remodel freshly deposited bone [7,139], while osteomalacia is abnormal osteoid mineralization that affects bone during its remodeling so unmineralized bone matrix accumulates in all parts of the skeleton [139,140].

In humans, nutritional rickets continues to be reemerging as a disease problem across the world [140,141]. Human rickets is seen usually during infancy and at puberty; the periods of maximum growth [142]. Nutritional rickets has been reported in young dogs [139] and cats [134], although this disease is considered rare [7]. However, rickets can be caused by an inborn error in vitamin D metabolism in dogs and cats [138,143,144,145,146,147].

Vitamin D-dependent rickets type I (VDDR-I), also referred to as vitamin D 1α-hydroxylase deficiency, is an autosomal recessive disorder caused by a deficiency of the renal enzyme 1α-hydroxylase due to a defect in the gene encoding it. Affected animals have normal serum calcidiol concentrations and low calcitriol concentrations [137,147]. Vitamin D-dependent rickets type II (VDDR-II) can also occur in animals as a result of an end-organ resistance to calcitriol and it is usually a secondary defect in the gene encoding the VDR [137,138,145].

A case of VDDR-I in a cat was characterized by hypocalcemia, hyperphosphatemia and skeletal abnormalities such as abnormal bone calcification, delayed teething and hyperparathyroidism [147]. The authors reported low calcitriol concentrations, and a genetic analysis proved the VDDR-I diagnostic. Variations of VDDR-I was reported after that. Grahn et al. [148] related a case by CYP27B1 mutation that caused a feline vitamin D-dependent rickets type 1A (VDDR-IA), and Teshima et al. [149] identified and reported a genetic variant of CYP2R1 identified in a cat with vitamin D-dependent rickets type 1B (VDDR-IB). This change caused low serum concentrations of ionized calcium and calcitriol, and both bone demineralization and abnormal growth plaques were observed on radiographic examination. In all cases, oral administration of calcitriol improved the cat’s wellbeing [147,149]. Although VDDR-I has been reported in cats, there is little research in its prevalence. However, such studies are hard to do as the diagnosis requires genetic analysis.

VDDR-II has been reported in dogs and cats and is a disease characterized by hypocalcemia, secondary hyperparathyroidism, low bone mineralization, rickets and, in some cases, alopecia [138,145]. The affected animals received higher doses of calcitriol associated with calcium supplementation for treatment because it is more difficult to increase circulating calcium concentrations compared to cases of VDDR-I [138,145]. Despite the association of calcitriol and calcium, the animals affected by VDDR-II generally showed persistent hypocalcemia. Godfrey et al. [145] observed no change in plasma calcium concentrations in a cat with VDDR-II after the administration of 110 ng/kg/day of calcitriol for five months and 3410 mg/kg/day of calcium carbonate. Likewise, Levine et al. [138] found no increase in serum calcium concentrations in a dog diagnosed with VDDR-II after supplementation of 64 ng/kg/day of calcitriol and 64 mg/kg/day of calcium carbonate.

Osteomalacia, another metabolic bone disease, rarely causes clinical signs in dogs or cats. However, the inability to detect bone pain and the insensitivity of radiographs of domestic animals, suggest that this condition may be underdiagnosed. In humans, the incidence of this disease remains largely underestimated across the globe as well [7]. Osteomalacia is an adult disease that occurs after cartilage growth is ceased. In animals, the clinical signs are dependent, and vary, according to age, growth rate and activity levels. In mammals, the lesions are most prominent in the long bones at the sites of rapid growth. They yield under the weight of the adult body, twist and bend, and deformities are presented. Fractures are extremely common, besides muscle weakness in elderly patients [150]. The treatment should be reversed by vitamin D supplementation. Calcium is also helpful particularly in those with a poor calcium intake [115,125].

## 9. Nutritional Secondary Hyperparathyroidism

Nutritional secondary hyperparathyroidism (NSHP) is a common consequence of a nutritional deficiency in pets consuming unbalanced diets [18,151,152]. It can be induced by low calcium concentrations, excess dietary phosphorus even in the presence of optimal dietary calcium, or in association with vitamin D deficiency [20,153]. Dietary vitamin D deficiency can result in NSHP both directly and indirectly. As previously discussed, the calcitriol receptor mRNA is highly expressed in the parathyroid gland [107,108] so that vitamin D also directly influences PTH secretion. Low vitamin D intake results in a decrease in circulating concentrations of calcitriol, which causes an increase in PTH synthesis, since one of the functions of calcitriol is to decrease PTH synthesis by suppressing the growth of parathyroid gland cells due to decrease in growth factors (transforming growth factor—epidermal growth factor receptor growth loop), and through the increase of inhibitors of cell growth (cyclin-dependent kinase inhibitors p21 and p27) [7,19].

Moreover, when low vitamin D dietary concentrations are available, there is a decrease in calcium absorption, which may result in hypocalcemia [154,155]. Low circulating calcium concentrations can stimulate the calcium receptors in the parathyroid gland leading to the production and secretion of PTH [20], which characterizes an indirect role of vitamin D in the development of NSHP. As a result of an excess of circulating PTH the number of osteocytes and osteoclasts increases, and calcium mobilization from bone to the extracellular fluid compartment occurs [154,156].

Few studies have reported NSHP in dogs and cats. Usually, the development of this change has been associated with the consumption of full meat-based diets by young animals due to the high calcium demand for bone growth [20,152,157]. In a report of six kittens affected by NSHP, they received a diet that had low calcium and phosphorus concentrations. Four of the six cats had been fed a full meat-based diet and the other two animals were fed a homemade diet containing only beef/chicken and rice. Some of them had severe osteopenia, which resulted in spontaneous fractures or excitation, muscle twitching or seizure. Four cats had clinical recovery after consuming a balanced diet, parenteral calcium gluconate and cage rest, while the other two cats had to be euthanized because of progressive neurological deficits secondary to spinal fractures [157]. More recently, a case was reported in which a six-month-old female dog with a history of lameness and difficulty in walking, and fed a nutritionally unbalanced homemade diet, developed NSHP. The authors reported that switching to suitable food that met nutritional recommendations was sufficient to promote the animal’s recovery [152].

Morphological changes in bones due to calcium mobilization makes the bones poriferous and prone to fracture. Mature bone replacement by fibrous tissue can lead the animal to develop fibrous osteodystrophy, which affects especially the jaw bones [7,151,152]. In a case report by de Fornel-Thibaud et al. [151], a six-year-old, spayed female rottweiler who consumed a homemade diet deficient in calcium and vitamin D developed facial enlargement from swelling of the maxilla and mandible, characterizing the rubber jaw syndrome. On radiographic and tomodensitometric examinations of the skull, severe osteopenia of the skull bones was observed. The animal had extremely low serum calcidiol concentrations (<0.7 nmol/L; reference range 19 to 90 nmol/L) and high plasma PTH concentrations (104.6 pmol/L; reference range 2 to 13 pmol/L). There was clinical improvement when switching to an adequate diet, with increased skull mineralization.

The treatment of animals with NSHP includes a complete and balanced diet, but sometimes it is necessary for the addition of calcium supplements. Cage rest is also important for the first few weeks of treatment in order to decrease the risk of bone fractures. The prognosis for noncomplicated cases is good and usually dietary correction normalizes body levels of calcium, phosphorus and PTH. On the other hand, complicated cases evolving spinal fractures, or more severe bony changes, support a bad prognosis [20,157].

## 10. Vitamin D Toxicosis in Small Animals

Signs of acute vitamin D toxicosis are related to the development of hypercalcemia when serum total calcium concentrations exceed 18 mg/dL, and serum ionized calcium concentrations are greater than 1.40 mmol/L. Clinical signs include polydipsia and polyuria due to difficulty in concentrating urine and stimulation of the thirst center, anorexia, vomiting and constipation as a consequence of decreased excitability of gastrointestinal smooth muscle and, finally, decreased neuromuscular excitability may cause weakness, depression, muscle twitching and seizures.

On the other hand, smaller but chronic intake of higher quantities of vitamin D (exceeding by 100 times de NRC recommended) are known as a cause of osteochondrosis and retarded bone remodeling in growing giant breed puppies like Great Danes, who do not have tight control of mineral deposition in large bones compared to smaller breeds [35,36,37].

Until now, cases of hypervitaminosis D are apparently rare and experimentally-induced, since the inclusion of this nutrient is controlled during pet food fabrication, although, in 2005, Mellanby et al. [21] reported two clinical cases of accidental and nutritional hypervitaminosis in dogs consuming a specific diet which was found at the manufacturing plant to be due to a human error that caused the over supplementation of vitamin D in the diet in which it should have been 80 IU/100 g but was 92.2 IU/g instead; over 100 times higher than the stated in manufacturer’s datasheet. The food was recalled from the market.

In both cases, dogs presented at the veterinary hospital with pyrexia, inappetence, polydipsia, polyuria, lethargy, a stiff gait, marked ionized calcium and total hypercalcemia. Palpation of both carpi demonstrated marked soft tissue swelling in one of them and flexion of both carpi caused considerable discomfort to the dog whose radiographs of the left and right carpi, elbows and stifles revealed multiple joint effusions. The consumption of the same diet indicated nutritional involvement. The dog from case one was treated only with intravenous saline solution for 24 h and sent home. The owners considered the dog to be generally free of clinical signs by day 180. On the other hand, the dog from case two received home oral steroid treatment associated with antibiotics and the owners reported that its condition was much improved and exercising normally by day 16.

In both cases, the food with vitamin D excess was withdrawn, ionized calcium was close or within the reference range by the second week after the first visit to the hospital. However, 180 days after, plasma concentrations of 25(OH)D were above the reference range, possibly due to its long half-life (29 days approximately), and may be why hypercalcemia signs persist for a while even when vitamin D was promptly taken out from diet [158].

As the etiology of vitamin D toxicosis is related to hypercalcemia, methods that inhibit bone resorption are the best alternative for treatment. In this scenario, intravenous injections of bisphosphonates are the best alternative, already described for humans and dogs [158,159]. It is important to consider that the approach to treating vitamin D toxicosis depends not only on the severity of hypercalcemia but also on the period of development (acute vs. chronic). Asymptomatic patients with mild hypercalcemia and with normal Ca × P product may not require immediate treatment, while individuals with severe acute increase in Ca levels may require more aggressive treatment. Accidental nutritional vitamin D toxicosis was reported in cats [22,23,24] and in those cases, respiratory discomfort was an important clinical sign among them due to soft tissue calcinosis. All of them were treated according to their symptoms, but only in a study by Crossley et al. [24], was bisphosphonate used and had a good clinical outcome. It is worth mentioning that in the study by Crossley et al. [24], of the three cats intoxicated with vitamin D and with hypercalcemia, pamidronate was administered in only one of them, since conventional therapy for hypercalcemia (fluid therapy, furosemide, prednisone) had no effect on circulating calcium concentrations, unlike in the others two cats, who had a good response to conventional therapy. An important observation of the before mentioned study is the fact that the cats were not made ill by a single food with inadequate amounts of vitamin D, but they received, as part of their diet, complimentary food in addition to dry extruded food, such as canned food and cat milk daily, and these were responsible for the reported vitamin D toxicosis. In this study, analysis was performed of cholecalciferol and ergocalciferol concentrations in the three foods consumed by the cats and all of them had vitamin D concentrations above the declared amount of added vitamin D. The canned food had vitamin D concentrations about 11 times higher than nutritional limit, and both dry food and kitten milk had vitamin D concentrations above the legal limit for declared additives. This is clearly striking and of great importance since these foods administered to patients were commercial foods.

The causes of hypervitaminosis D are not always nutritional, and the most common one is cholecalciferol rodenticide described in dogs [25,26,27,28]. Granulomatous diseases like sarcoidosis and Wegener granulomatosis can also produce hypercalcemia through the unregulated production of calcitriol, which is called metabolite-mediated hypercalcemia [160]. In addition, there are other possible causes of vitamin D toxicity, such as accidental consumption of plants containing calcitriol glycosides such as jessamine [30] and topical ointments based on vitamin D analogues such as calcipotriol and maxacalcitol, which are used for psoriasis treatment [31,32,33,34].

## 11. Vitamin D and Its Nonclassical Actions

The nonclassical actions of vitamin D were not a focus of this review. However it is important to remember that vitamin D is also related to numerous functions not associated with bone metabolism [3,44]. From 1983, when the presence of vitamin D receptors in human immune cells was discovered, research on the unrelated effects on bone metabolism of vitamin D intensified, and it was discovered that cells in almost all body tissues express vitamin D receptors (3).

In small animals has been demonstrated an association between low vitamin D circulating concentrations and diseases not associated with bone metabolism, such as: cancer [161,162,163,164], congestive heart failure [165,166], gastrointestinal diseases [167,168,169,170,171], acute pancreatitis [172], acute polyradiculoneuritis [173], CKD [161,174,175,176,177] and infectious diseases [178,179,180,181]. It has also been demonstrated in hospitalized cats that when 25(OH)D was represented as a categorical variable, cats with 25(OH)D circulating concentrations in the lower tertile had a higher risk of mortality compared to cats in the reference category and the middle tertile [182]. In a study with hospitalized dogs in a critical condition, it was observed that 25(OH)D serum concentrations at the time of diagnosis was a predictor of mortality rate for dogs with chronic enteropathy [171].

In addition, in-vitro antineoplastic effects of calcitriol have been shown in various types of canine tumors, including osteosarcoma [183], squamous cell carcinoma [184], prostatic epithelial tumor [185], anal sac adenocarcinoma [186], mammary gland cancer [187] and mastocytoma [188]; as well as in vivo antineoplastic effects in dogs with mastocytoma [188].

It is not yet clear whether low vitamin D status is the cause or consequence of the diseases mentioned above, with evidence supporting both hypotheses [3].

## 12. Conclusions

It is concluded that dogs and cats have different vitamin D metabolisms compared to other mammal species. Little is known about the vitamin D requirements for dogs and cats, especially concerning adult animals and females in the reproductive phase. New trends in dog and cat nutrition, such as a homemade diet, may increase the risk of developing vitamin D deficiency. Vitamin D poisoning is not common in dogs and cats, but it can occur as a result of errors in the formulation of commercial foods, accidental consumption of rodenticides, plants containing calcitriol glycosides and topical ointments based on vitamin D analogues.

## Figures and Tables

**Table 1:** Minimum and maximum recommendations of dietary cholecalciferol for adult dogs and cats according to scientific literature.

	Minimum		Maximum	
	Dog	Cat	Dog	Cat
NRC (2006) (IU/Mkcal)	110 ^a^/136 ^b^	56 ^a^/70 ^b^	800	7520
FEDIAF (2020) (IU/Mkcal)	138 ^c^/159 ^d^	62.5 ^e^/83.3 ^f^	800	7500
AAFCO (2018) (IU/Mkcal)	125	70	750	7520

^a^ Adequate intake; ^b^ recommended allowance; ^c^ 110 kcal × (kg of body weight)^0.75^ as fed; ^d^ 95 kcal × (kg of body weight)^0.75^ as fed; ^e^ 100 kcal × (kg of body weight)^0.67^ as fed; ^f^ 75 kcal × (kg of body weight)^0.67^ as fed. NRC = National Research Council; FEDIAF = European Pet Food Industry Federation; AAFCO = American Feed Control Officials; IU = international units; Mkcal = megacalories.

**Table 2:** Minimum and maximum recommendations of dietary cholecalciferol for growing puppies and kittens according to scientific literature.

	Minimum		Maximum	
	Dogs	Cats	Dogs	Cats
NRC (2006) (IU/Mkcal)	110 ^a^–136 ^b^	- ^a^/56 ^b^	800	7520
FEDIAF (2020) (IU/Mkcal)	125 ^c^/138 ^d^	70	800	7500
AAFCO (2018) (IU/Mkcal)	125	70	750	7520

^a^ Adequate intake; ^b^ recommended allowance; ^c^ Late growth (>14 weeks); ^d^ Early growth (<14 weeks). NRC = National Research Council; FEDIAF = European Pet Food Industry Federation; AAFCO = American Feed Control Officials; IU = international units; Mkcal = megacalories.
